# Feasibility and Acceptability of a Mobile Phone App Intervention for Coping With Cancer as a Young Adult: Pilot Trial and Thematic Analysis

**DOI:** 10.2196/25069

**Published:** 2021-06-11

**Authors:** Hanneke Poort, Annelise Ryan, Katelyn MacDougall, Paige Malinowski, Anna MacDonald, Zach Markin, William Pirl, Joseph Greer, Karen Fasciano

**Affiliations:** 1 Department of Psychosocial Oncology and Palliative Care Dana-Farber Cancer Institute Boston, MA United States; 2 HTD Health New York, NY United States; 3 Center for Psychiatric Oncology and Behavioral Sciences Massachusetts General Hospital Boston, MA United States

**Keywords:** mobile phone, mobile phone application, cancer, feasibility

## Abstract

**Background:**

Many young adult patients do not receive adequate psychosocial services to help them cope with cancer.

**Objective:**

This study aims to assess the feasibility and acceptability of a smartphone app (*iaya*) intervention that was designed to create an engaged community of young adult patients and help them learn emotional coping skills.

**Methods:**

For this single-group pilot trial, 25 young adult patients aged 18-39 years who were receiving active cancer treatment were asked to use the *iaya* app for 12 weeks. To collect app use data, we used Mixpanel, an analytics platform for apps. Feasibility was assessed through rates of app sessions and the number of coping exercises engaged, and intervention acceptability was evaluated by using an app usability questionnaire and through qualitative interviews at study completion. We collected patient-reported outcome data at baseline and at week 12 to explore self-efficacy for coping with cancer, self-efficacy for managing emotions, perceived emotional support, and quality of life.

**Results:**

Baseline patient-reported outcome data indicated that participants scored relatively low on perceived emotional support but reasonably high on self-efficacy for coping with cancer and managing emotions as well as quality of life. Participants had a mean of 13 app sessions (SD 14) and 2 coping exercises (SD 3.83) in 12 weeks. Only 9% (2/23) of participants met our combined feasibility definition of ≥10 app sessions and ≥3 coping skills from different categories. The participants’ mean usability score was 73.7% (SD 10.84), which exceeded our predefined threshold of ≥70%, and qualitative feedback was generally positive.

**Conclusions:**

Although perceived acceptable by patients, the *iaya* smartphone app did not meet the a priori feasibility criteria as a stand-alone app intervention. Future studies should screen participants for unmet coping needs and consider integrating the app as part of psychosocial care for young adult patients.

## Introduction

### Background

Young adults (aged 18-39 years) experience a unique set of psychosocial needs while receiving treatment for cancer. Young adulthood, a phase of life critical to development, is characterized by establishing identities, negotiating independence from parents, completing education, starting a career, and making life decisions about relationships and family [1]. A cancer diagnosis can interrupt these milestones and severely alter a young adult’s life and affect their psychological well-being [2,3]. Moreover, young adults are at a unique stage in their emotional, cognitive, and social development, and many young adults with cancer report high levels of cancer-related distress and other psychological issues, such as isolation, anxiety, and depression [4].

The National Comprehensive Cancer Network guidelines call for developmentally appropriate psychosocial support services for young adults with cancer. These services must be flexible in approach; provide age-appropriate information on important topics such as financial health, fertility, work, body image, and sex; and recognize the importance of peer support [5]. Despite the existing body of research in this field and the subsequent recommendations, a substantial proportion of young adults (41%) report unmet needs for psychosocial support during the first year following diagnosis [2]. Failure to meet such psychosocial support needs is associated with increased distress in this population [6]; therefore, providing these services is of utmost importance in the care of young adults with cancer.

Several factors may interfere with young adults’ access to or utilization of psychosocial care services. One barrier is the lack of awareness among the general adult cancer population about the available services. In 2010, the National Health Interview Survey reported that 90% of patients did not know that psychosocial services existed or that they were available to them [7]. Furthermore, young adults can be difficult to engage in psychosocial services because of the stigma of accessing mental health services [8]. In addition, young adults typically have lives with multiple demands outside of coping with cancer, which may reduce a young adult’s ability and willingness to attend on-site peer support activities and psychosocial care visits [9]. More specifically, young adults may be unable or less willing to participate in in-person activities that address their psychosocial needs because of ill-health, treatment side effects, and the considerable effort required to manage and complete treatment [10,11]. Therefore, it is important to explore interventions that promote psychosocial support services that young adults can easily access and incorporate into their lives without jeopardizing competing priorities.

Solutions involving technology and social media show promise for delivering age-appropriate psychosocial care to young adults with cancer, as they can also eliminate some of the access barriers. As of 2019, 96% of young adults aged 18-29 years reported having a smartphone, and 100% of millennials aged 23-38 years said that they used the internet [12,13]. In addition, the majority of young adults are comfortable using social media platforms, and over half of all 18-29–year-olds who are on the web access these types of sites daily [14]. Specifically, within the cancer sphere, young adults have expressed interest in smartphone apps and social media interventions aimed at providing mental health care and peer support [15,16]. Other investigators have also recognized the potential of delivering cancer-related care via smartphone app. However, most cancer-related apps aim to raise awareness and provide information, whereas a smaller subset targets prevention, early detection, management of cancer, and social networking [17,18]. Very few studies on cancer-related apps have attempted to explore the effectiveness and utility of delivering psychosocial care, and even fewer studies have explored the delivery of age-appropriate psychosocial care for young adults with cancer [19,20].

### Objective

In this work, we describe the pilot testing of a smartphone app intervention (*iaya*) in young adults who were receiving active cancer treatment. The *iaya* app was designed with input and feedback from young adults to build an engaged community, to develop coping skills, and to encourage personal development. The goal of this study is to assess the feasibility (defined as number of app sessions and coping skills engaged) and acceptability of the *iaya* app intervention among young adults with cancer.

## Methods

### Study Setting and Participants

We conducted a single-arm pilot study in the lymphoma, leukemia, breast, melanoma, sarcoma, gastrointestinal, and neuro-oncology clinics at the Dana-Farber Cancer Institute in Boston, Massachusetts, between November 11, 2019, and March 13, 2020. The eligibility criteria were as follows: had a diagnosis of cancer, were receiving active cancer treatment, were under the care of a Dana-Farber oncologist, aged 19-39 years, had access to a smartphone (iOS or Android), and had a willingness to use the *iaya* app and complete study surveys as well as a qualitative exit interview. Exclusion criteria included the following: an inability to provide informed consent in English and cognitive or neurological impairments that might preclude study participation, as evaluated by the research study staff or oncology provider. We aimed to enroll 30 participants for this pilot feasibility trial over a 6-month enrollment period but suspended recruitment 2 months earlier because of halted research operations related to the COVID-19 pandemic.

### Enrollment

We conducted a limited review of clinic schedules to identify potentially eligible patients. A research staff member (AR) contacted the patient’s oncology provider to confirm eligibility and request permission to approach the patient at an upcoming clinic visit. Patients approved for approach for enrollment were invited by a research staff member to enroll in the study. Patients were informed during the consent process that the app was freely available outside of study participation, as the app was first launched as a clinical tool on April 6, 2019, as part of the Dana-Farber Young Adult Program. Participants were specifically informed about the data that would be collected from their phone, the methods used to secure and encrypt these data, and the information that would be used for the study. All participants provided written informed consent for all the study procedures. The participants did not receive financial incentives for completion of study procedures. The study was approved by the Institutional Review Board of the Dana-Farber/Harvard Cancer Center.

### Intervention

The *iaya* intervention was a smartphone app intervention designed to improve the psychological care for young adults with cancer, and it consisted of several components. Clinical content for the *iaya* app was developed by mental health clinicians specializing in the care for young adult patients with cancer (KM and KF). We sought to accomplish our goal of improving young adult–specific psychological care by providing users with an app that included a combination of psychoeducational resources, coping skills training, and the opportunity to connect and share personal content with peers. All these components have theoretical underpinnings, including the sense of coherence theory [21,22], coping theory [23], and social support theory [24,25]. In addition, the opportunity to connect and share personal content with peers was also endorsed as a cancer-specific need by our app development stakeholder group (refer to the *App Development* section). The *iaya* app also included a geolocation feature that users could opt for if they were at Dana-Farber and which was meant to serve as a *virtual waiting room* to connect with other young adults with cancer on the app and at Dana-Farber at the time.

**Figure 1:**
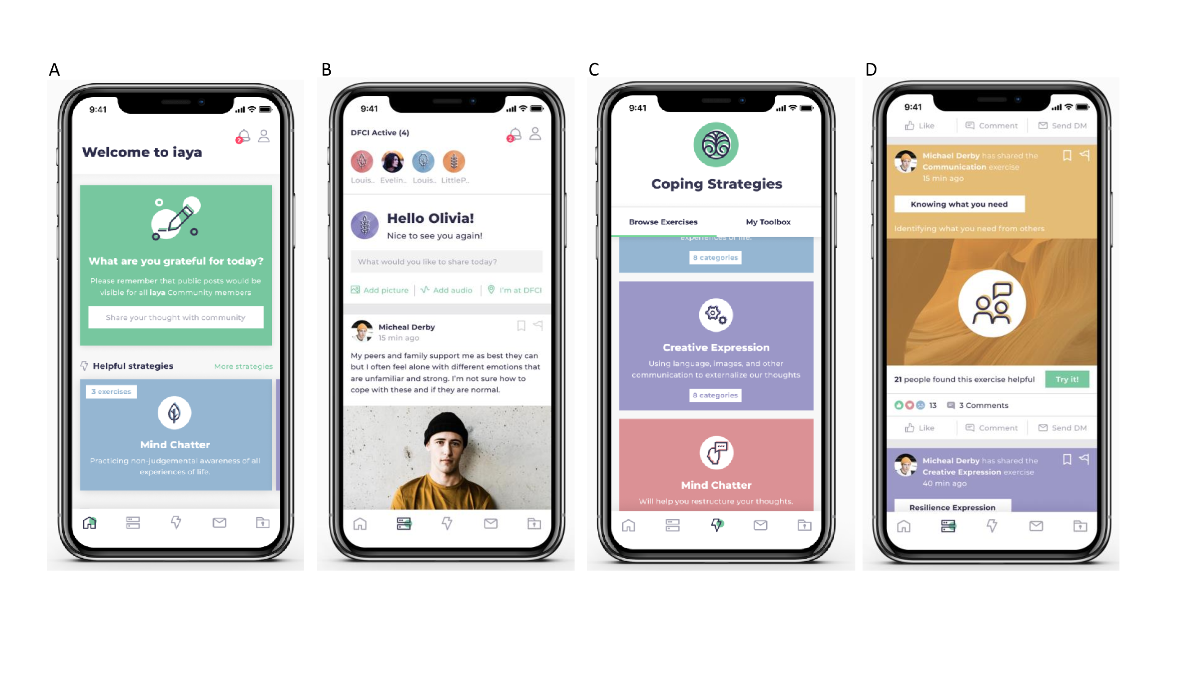
Features and interface of the iaya app. Main app features displayed on a (fictive) user’s smartphone, as follows: iPhone smartphone screens displaying the home page with a community question (A) and the community feed with active users on the web (B) and the option to check into the virtual waiting room through the geolocation feature “I’m at DFCI.” iPhone smartphone screens displaying the community feed with a user’s shared output from a coping exercise (C) to which users can respond by liking, commenting, direct messaging, or trying it themselves and the coping strategies section (D) in which users have the option to browse exercises or go to “My Toolbox,” where they shortlisted previously completed exercises for easy access. DFCI: Dana-Farber Cancer Institute.

Figure 1 displays the interface and 5 main features of the app: the home page, community feed, private messaging, private feed, and coping exercises section. The dynamic *home page* had a rotating community question that users could respond to; a shortcut to the coping exercises; and the YAP Daily Post, which featured a different young adult resource every day. Notifications could be enabled or disabled based on an individual user’s preference. If left enabled, users would receive a notification every time the community question rotated on the home page, which was 2-3 times a week. Similarly, users could either create a profile with their real name or create a username based on their individual preference. In the *community feed*, users could post information on a public forum and comment or react to other users’ posts. If users wanted to connect with other users privately, they were able to send direct messages, in the *private messaging* section, to individuals that they met on the community feed. The *private feed* served as a space for users to note their thoughts and save meaningful information they found on the community feed. The *coping exercises* section in the app featured exercises from evidence-based therapies used in clinical practice to help treat people with anxiety and mood difficulties (ie, cognitive behavioral therapy, dialectical behavior therapy, acceptance and commitment therapy, and mindfulness-based stress reduction) as well as strategies that help facilitate emotional resilience.

In the coping exercises section, users had access to 131 unique exercises that were divided into 6 categories: Values (identifying things we care most about), Communication (clear assertive communication to optimize self-advocacy and support from others), Let It Be (building skills to limit struggles with aspects of life we cannot control), Mind Chatter (help restructure your thoughts), Take Action (feel fully connected to your body, especially when you feel triggered by anxiety or worry), and Expression (using language, images, and other communication to externalize and express thoughts). Textbox 1 provides a more detailed description of these categories and their content. Coping strategies included both educational information and active exercises to practice the strategy. The active exercises allowed the user to generate an output to share on the community feed.

**Coping exercise categories and content.**

**Values: “Identifying the Things We Care Most About”**

- Exercises to help the user concentrate on ways to define and focus on what is important to them and how this can help with enhancing positive emotions and shift away from negative emotions. Users will explore the relationship between values and goals. Finally, users are asked to strengthen their identity outside of their illness by focusing on values and redefining goals to meet values.

**Communication: “Clear Assertive Communication to Optimize Self-advocacy and Support From Others”**

- Exercises designed to help young people enhance positive strategies for adjusting to needing help and defining and asking for what they need. This section also includes exercises for skills in setting limits and boundaries for self-care as well as improving open communication to enhance social support. In addition, users are asked to practice communication that conveys thanks and gratitude. Finally, users are coached through strategies for disclosure about cancer.

**Let It Be: “Building Skills to Limit Struggles With Aspects of Life We Can’t Control”**

- Exercises aimed at identifying and normalizing emotions, expression of emotions, and defining what can and cannot be controlled. Users learn more about acceptance and accepting emotions as well as being intentional with emotions.

**Mind Chatter: “Will Help You Restructure Your Thoughts”**

- Exercises aimed at restructuring thoughts that may be cognitive errors. Common cognitive errors are highlighted and include thinking filter, blaming, jumping to conclusions, control myths, emotional reasoning, and personalization. For each of these, users are asked if they can relate to the error, and if so, the user is asked to review examples, restructure a thought that is presented to them, and come up with their own example and a restructured solution.

**Take Action: “Feel Fully Connected to Your Body, Especially When Triggered by Anxiety or Worry”**

- Exercises include behavioral strategies that reduce the stress response and enhance mindfulness. Some of these strategies promote present moment awareness, whereas other guided imagery strategies take the user into a created visual image that enhances relaxation. Basic breathing is also addressed in this category, as are progressive muscle relaxation, grounding, and mindful action techniques.

**Expression: “Using Language, Images, and Other Communication to Externalize Our Thoughts”**

- Exercises in this category provide the user with prompts for posting written, visual, or auditory contributions to express themselves around young adult’s identity development in general or to the challenges and impact of cancer. Users are provided with prompts to promote expression of resilience-based principles such as gratitude, defining successes, meaningful actions, and priorities.

### App Development

We developed the *iaya* app as a stand-alone intervention with input from mental health clinicians of the Young Adult Program at Dana-Farber (KF and KM), other psychosocial clinicians at Dana-Farber, and engineers from HTD Health (New York, New York), with funding from the Oak Foundation. In addition, we had 8 stakeholder meetings with young adult patients between November 2016 and March 2019. Three early meetings (2016-2017) were focused on soliciting general thoughts about and preferences for technology solutions for providing psychosocial care to young adults. An additional 5 meetings (2018-2019) were specifically focused on the user testing of prototypes and beta versions of the app. After the launch of the app in April 2019 (ie, 6 months before opening the feasibility study), we invited 9 young adult patients from the stakeholder meetings to remain involved as *super users*. We encouraged these *super users* to continue to beta test and provide real-time user feedback through an in-app feedback and bug reporting tool for smartphone apps (Instabug). In addition, we shared suggestions with them via weekly emails for 2 months on how to increase user engagement on the app, such as responding to other users’ contributions and posting content.

### App Use

The *iaya* app is a microsocial network that exposes a native smartphone app for iOS and Android to young adult patients with cancer and includes a separate web interface for community and content management. All data are encrypted while in transit and stored on an encrypted database server provided by the Partners Health System. All data were collected and stored in a Health Insurance Portability and Accountability Act–compliant manner. We used the Mixpanel analytics platform to collect data on participants’ use behavior. We gathered information unique to users, yet fully deidentified, to understand the frequency of app use. We also recorded the number of coping exercises engaged. Deidentified use data were sent directly from a user’s mobile device to Mixpanel, where it was stored with a unique and anonymous identifier for later analyses.

### Procedures and Reminders

Participants were asked to download the freely available *iaya* app and recommended to use the app ≥3 times per week. During the first 3 weeks of study participation, a research assistant with a master’s degree in public health (AR) conducted weekly phone calls to see if participants had any technical issues with accessing or using the app. Thereafter, the participants received one more phone call at 6 weeks. If phone calls were unanswered, a voicemail message was left with the instruction to return the call, in case of any technical difficulties with the app. In addition to technical support, these phone calls also served as reminders of the *iaya* app. This hybrid approach was adopted in an attempt to minimize early intervention dropouts and to overcome poor uptake of the smartphone app.

### Revisions and Updating

No major changes to features or content were made to the app during the research study. At the time of study commencement (November 11, 2019), participants downloaded the 1.0.20 version of the *iaya* app. Early in the study, an app update was required to ensure correct data collection by Mixpanel. We released version 1.0.21 on December 11, 2019, at which point the research assistant contacted the 12 participants actively enrolled in the study to update their app to the most recent version. All but one participant updated their app to the new version, which resulted in a loss of use data from this participant. Three months into the study, we required another update because data collection to measure the number of app sessions through Mixpanel was incompatible with the Android platform. We released version 1.0.22 on March 16, 2020, to correct this unforeseen issue, which was downloaded by 5 participants. Finally, version 1.0.23 was released 1 week later, with minor enhancements to improve the *currently online* feature of the app, and this version was downloaded by 3 participants during the study period.

### Measures

#### Demographics

Participants completed a basic sociodemographic questionnaire at enrollment (eg, age, marital status, children, race, education, employment, and religion). Clinical information, including primary disease site, time since diagnosis, and current cancer treatment, was extracted from the electronic health record.

#### Feasibility

Feasibility was defined as ≥75% of participants with ≥10 app sessions (ie, a user opens the app and has it open for at least 10 seconds before the app is closed or moved to the background) and ≥3 coping skills opened from different categories over a period of 12 weeks. We corrected the auto-populated Mixpanel app session data to eliminate multiple app sessions within a 5-minute window.

#### Acceptability

Acceptability was defined as ≥70% mean app usability score derived from the app usability questionnaire that participants filled out upon study completion. This questionnaire was adapted from the 10-item System Usability Scale [26] to a simplified 6-item scale. Items are scored on a 5-point Likert scale (1=strongly disagree; 5=strongly agree), and each item’s score contribution ranges from 0 to 4. Sum scores were converted to a 0-100 range; higher scores indicated a higher perception of usability. Scores ≥70% can be considered to have good usability. In addition, patients participated in qualitative exit interviews with a member of the study team (AR) to elicit feedback on their experiences with the app intervention. The interviews were audio-recorded and transcribed.

#### Patient-Reported Outcomes

We included 4 measures to explore self-efficacy for coping with cancer, self-efficacy for managing emotions, perceived emotional support, and quality of life at baseline and 12 weeks. The study data were collected outside of the smartphone app and were managed using REDCap (Research Electronic Data Capture) hosted at Partners Healthcare.

The Cancer Behavior Inventory-Brief Version (CBI-B) is a 12-item questionnaire that assesses self-efficacy regarding different coping skills in the context of cancer (eg, maintaining a positive attitude, asking physicians questions, seeking consolation, and coping with physical changes) [27,28]. Items are scored on a 1-9 Likert scale (1=not at all confident; 9=totally confident). We calculated a total summed score (range 12-108), with higher scores indicating greater self-efficacy for coping, which is associated with better adjustment to cancer.

We used the 8-item Patient-Reported Outcomes Measurement Information System (PROMIS) Short Form v1.0-Self-Efficacy for Managing Emotions 8a to assess a participant’s level of confidence in managing symptoms of anxiety, distress, discouragement, disappointment, and negative feelings [29,30]. Items are scored on a 1-5 Likert scale (1=I am not at all confident; 5=I am very confident). We also used the 8-item short form PROMIS Short Form v2.0-Emotional Support 8a to assess perceived feelings of being cared for and valued as a person [31]. Items are scored on a 1-5 Likert scale (1=never; 5=always). For both PROMIS measures, we calculated a summed score and converted the total raw score into a T-score for each participant [32]. A higher PROMIS T-score represents more of the concept being measured (ie, greater self-efficacy for managing emotions and more emotional support). The standardized T-score has a mean of 50 (SD 10), indicating the average for the general chronic condition population in United States.

The 27-item Functional Assessment of Cancer Therapy-General (FACT-G) was used to assess the general quality of life [33]. Items are scored on a 0-4 Likert scale (0=not at all; 4=very much). Subscale scores were summed to obtain a FACT-G total score (range 0-108), with higher scores indicating a better quality of life.

### Data Analysis

Descriptive statistics were used to describe the characteristics of our sample and summarize patient-reported outcome data at baseline and at 12 weeks. In addition, we used an inductive (content-driven) approach to code and analyze the exit interviews using thematic analysis [34]. First, the study team members HP and AR performed open coding and memoing to evaluate the transcripts and repeatedly reviewed transcripts line by line to identify text related to participants’ perceptions of the *iaya* app and suggestions for improvement. Next, these study team members (HP and AR) met regularly to systematically review and discuss coded contents to identify emergent themes and patterns and synthesize data across themes, both within and across participant types (eg, users vs nonusers of coping strategies).

## Results

### Overview

**Figure 2:**
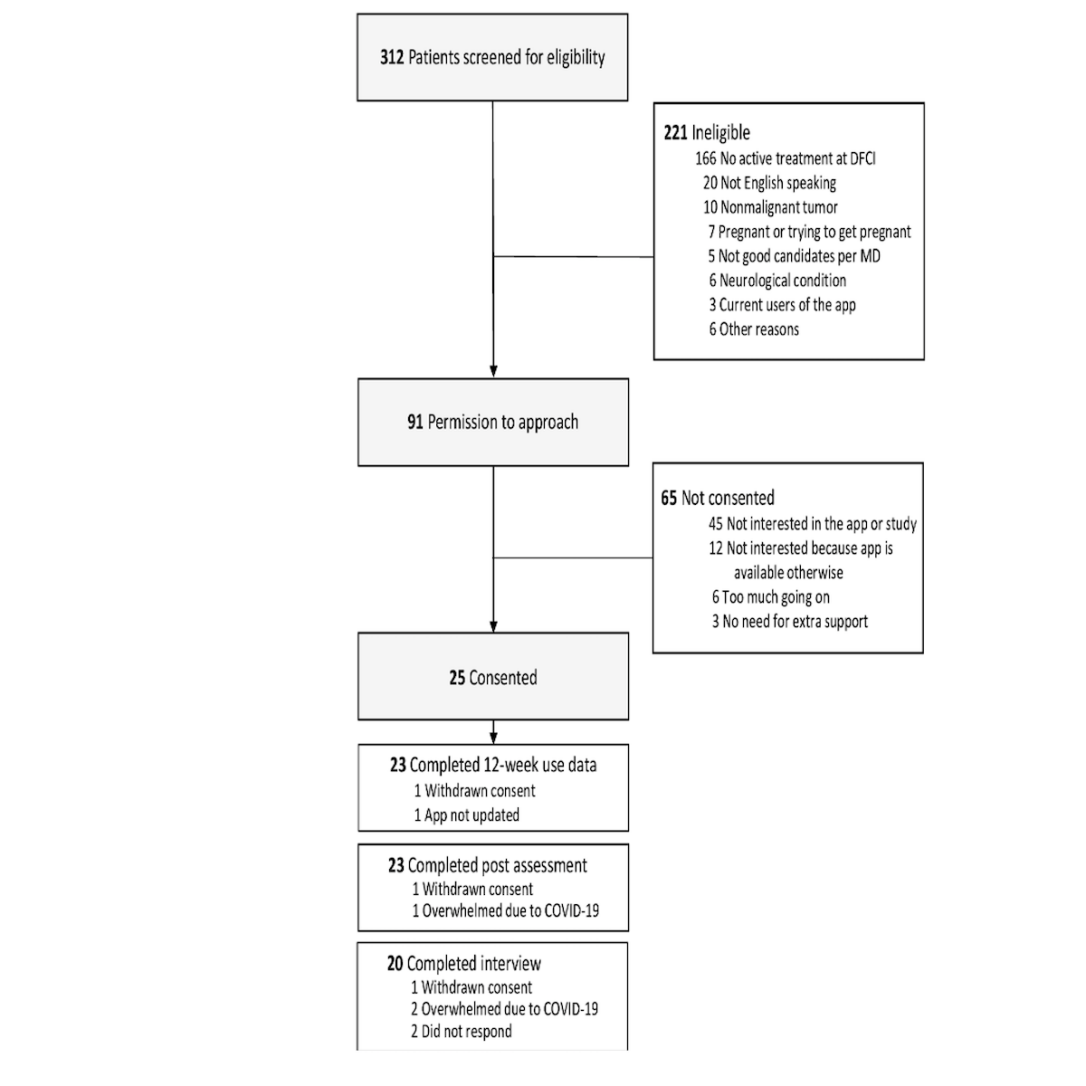
Flowchart of screening and enrollment. DFCI: Dana-Farber Cancer Institute; MD: medical doctor.

We identified 97 potentially eligible patients during the 4-month enrollment period (Figure 2). Of these, 5 were deemed inappropriate by their treating oncologist for the following reasons: *bad timing for the patient* (n=1), *too distressed* (n=1), and *patient disposition* (n=3). Among the 92 patients who were approached to participate, 25 (27%) provided consent and were enrolled in the study. The 3 most common reasons for declining study participation were not interested in participating in the study, not interested in study because the app was available otherwise, and the patient had too much going on at the time. As displayed in Table 1, the mean age was 28 years (SD 5); 14 participants were female, 14 were married, and 5 identified themselves as Black individuals or other people of color. A total of 23 participants had a college or advanced degree, and 13 participants were employed.

**Table 1:** Participant characteristics (N=25).

Characteristic		Value
Age (years), mean (SD)		28 (5)
**Sex, n (%)**		
	Female	14 (56)
	Male	11 (44)
**Marital status, n (%)**		
	Married	14 (56)
	Single, never married	7 (28)
	Noncohabitating relationship	3 (12)
	Divorced	1 (4)
Any children, n (%)		9 (36)
**Race, n (%)**		
	White	20 (80)
	Black	3 (12)
	Other	2 (8)
**Ethnicity, n (%)**		
	Non-Hispanic	23 (92)
	Hispanic	1 (4)
	Prefer not to answer	1 (4)
**Education, n (%)**		
	High school	1 (4)
	College student	2 (8)
	College graduate or advanced degree	22 (88)
**Employment, n (%)**		
	Employed	13 (52)
	Disabled	8 (32)
	Caring for home or family	2 (8)
	Student	2 (8)
**Primary disease site, n (%)**		
	Lymphoma	9 (36)
	Breast	5 (20)
	Sarcoma	5 (20)
	Neuro	3 (12)
	Gastrointestinal	1 (4)
	Leukemia	1 (4)
	Melanoma	1 (4)
**Stage of disease, n (%)**		
	Early	11 (44)
	Late	12 (48)
	Not available^a^	2 (8)
**Time since diagnosis (years), n (%)**		
	0	16 (64)
	1-2	8 (32)
	≥3	1 (4)
**Cancer treatment, n (%)**		
	Chemotherapy	22 (88)
	Immunotherapy	2 (8)
	Radiation	1 (4)

^a^Acute lymphoblastic leukemia or T-lymphoblastic lymphoma (n=1) and myelodysplastic or myeloproliferative neoplasm (n=1).

### Feasibility

App use data were available for 23 participants. One participant had an older version of the smartphone app (1.0.20) that was incompatible with Mixpanel, and one participant completed only 3 weeks of the study period before withdrawing consent due to medical reasons. On average, participants had 13 app sessions (SD 14; range 1-50) in 12 weeks. A total of 48% (11/23) participants had ≥10 app sessions in 12 weeks. In addition, participants opened an average of 2 (SD 4; range 0-18) individual coping exercises. Two participants opened ≥3 coping skills from different categories. Thus, only 9% (2/23) of participants met our combined feasibility definition (ie, ≥10 app sessions and ≥3 coping skills from different categories over a period of 12 weeks).

### Acceptability

#### Overview

Upon completion of the 12-week study period, 23 participants completed the app usability questionnaire. One participant did not complete any surveys, and one participant withdrew consent due to medical reasons. The mean app usability score from 23 participants was 73.7%, which exceeded our predefined acceptability threshold of ≥70%.

The interviews indicated that participants liked the app’s overall design and features. For example, one patient said:

It looks pretty neat, and I can find what I’m looking for really fast, which is pretty good. I like it.

P4

Another participant noted:

It was pretty self-explanatory, easy to navigate through.

P12

The participants only had minor suggestions for technical or content improvements. Three themes emerged from the qualitative data and are described below. Additional illustrative quotes for each theme are provided in Textbox 2.

**Themes and illustrative quotes from exit interviews.**

**Social Support and Feelings of Isolation**

- “I can definitely see people are trying to encourage each other. That is great. I also see several people after they finish treatment, they still come back and provided support which is super nice.” (P4)
- “I think for me, the feeling of being alone in it was the biggest thing and then going into the app and seeing, I was just taken back at actually how many people my age are sick with it.” (P7)
- “I’m kind of like a shy person, so I didn’t really want to make the approach myself, but it was nice to see, I guess you perceive support from seeing other people going through similar struggles as you....I didn’t make a connection with someone but if I wanted to meet them in person, I guess that would have been nice.” (P11)
- “The solidarity was very important. I didn’t necessarily post or message anybody but reading through what people wrote and knowing that other people are going through different things is very useful.” (P15)
- “My peers at home are not going through this. Just knowing that other people are going through this and that I’m going to be okay. That I have people out there, that there are other people rooting for me....It made me feel less alone.” (P19)

**Use Versus Nonuse of Coping Exercises**

- “I’ve mostly just used the coping mechanisms....A lot of the questions I felt calmed me down so that worked for me. They were pretty well done.” (P9, user)
- “Favorite feature of the app, definitely. It helped with communicating with myself, so to speak, and understand or gain greater clarity on some of the emotions with the coping strategies that help you walk through a process. I think just identifying and parsing out your feelings.” (P15, user)
- “Favorite feature, definitely the meditation and breathing exercises. I’ve been really stressed recently, and I’ve just been working on relaxation, meditation, so I really like those two of the exercises. They’ve been helping me.” (P19, user)
- “I wasn’t really that focused on looking at the coping things, because I had a friend who had cancer when she was younger, so she was a big part of asking for coping advice.” (P2, nonuser)
- “I just didn’t feel the need to [use coping exercises] because I have people to talk to and I feel like I’ve naturally been able to cope with it independently and with some help. I didn’t think I was going to get anything from it, to be honest.” (P8, nonuser)
- “I liked the coping strategies section too. I didn’t use it very much, but it seemed very straightforward and helpful for people who did need to use it....I’m kind of familiar with some of the coping skills. I see a therapist and also, I’m a psych student, and so I don’t think I needed them.” (P10, nonuser)

**Low User Engagement**

- “I think one of the tough parts is that, compared to other apps, there isn’t a lot of people....It’s tough to go frequently on the app if there’s not as many people.” (P3)
- “I forget to use it most of the time....This community now is pretty small so it would be great if the community is a little larger.” (P4)
- “I think it would be better if there were more people on it at a time. I don’t think there was many, I know you guys are just trying this out so it’s not like there’s as many people on it as I think would eventually be on the app.” (P17)
- “I kind of like went in and out. There would be sometimes where I would log on almost daily and then I would go weeks and not look at it. I just thought it was kind of sporadic, but I will say that that is the way that I am with most social media.” (P20)
- “I have been meeting with multiple therapists during this whole time and so I was having my needs met by my therapist and the app became a side thing.” (P22)

#### Theme 1: Social Support and Feelings of Isolation

The majority of patients indicated the community feed as their favorite feature, whereas only a minority named the coping exercises. Specifically, patients appreciated the opportunity to connect with peers, receive support, and read about their experiences. Patients felt less isolated knowing and seeing other people of their age dealing with cancer and reporting similar experiences. For example, one patient explained:

I thought it was very helpful because I was able to connect and message a few people who went through kind of the exact thing. So, it was a support that I didn’t get anywhere else because they had the advice that I needed that nobody else could give me.

P10

#### Theme 2: Use Versus Nonuse of Coping Strategies

Interestingly, several patients noted that despite not using the app intensively, they felt that they still received what they needed from it. For example, one patient said:

I liked that it was flexible. That you didn’t have to be on it all the time, that you could just check back in on it based on what you wanted to do.

P17

Most patients confirmed that they did not use the coping exercises that much, with many of them explaining that they already felt their needs in this area were being met. For those who engaged with this part of the app, the feedback was positive. For example:

A lot of the exercises I felt calmed me down, so that worked for me. They were pretty well done.

P9

#### Theme 3: Low User Engagement

Importantly, several participants noted that there were not many users on the app and not many people actively posted content, making the app less engaging and not offering as much opportunity for social connections. Ironically, many of these patients also confessed that they themselves were “more of a viewer than someone to add to things” (P24) on social media and that they were also using the *iaya* app that way. Patients reported varying preferences about the timing of the app in relation to their disease trajectory, with some stating that they found it most useful during active treatment, whereas others said they felt that time was too overwhelming and they preferred exploring the app further after completion of cancer treatment. Several patients suggested that a larger userbase and more turnover of information on the app would facilitate better engagement. However, a few patients also stated that they were hesitant to share content on the app because they did not want to burden other patients with their struggles. Finally, some participants expressed that they would appreciate connecting with patients with a similar diagnosis, because of the variation in treatment for different cancer types, or in a similar life stage, as there are still significant differences between young adults who are college students and those who are young professionals and/or parents.

When specifically asked about the potential negative emotional impact of using the app, only one patient said they felt some of the content shared by other participants triggered certain negative emotions. In addition, a few patients noted that it was sometimes difficult for them to read some of the content on the community feed, particularly when other patients were not doing as well as them. For example, one patient said:

Cancer isn’t always that nice to everybody, so then you go in the app and people have had really bad setbacks and so when you’re not having a great emotional day with your own that can be hard to read.

P20

### Patient-Reported Outcomes

Table 2 shows the baseline and 12-week scores for the patient-reported outcomes. The average CBI-B score among our sample was 84.88 (SD 14.66) at baseline and decreased to 75.74 (SD 14.43) at follow-up, which indicates relatively high self-efficacy for coping with cancer. For the PROMIS self-efficacy for managing emotions, the average T-score was 49.09 (SD 8.21) at baseline and 48.64 (SD 7.80) at follow-up, indicating that our sample had similar self-efficacy for managing emotions compared with the general chronic condition population. For the PROMIS emotional support, the average T-score was 55.24 (SD 7.11) at baseline and 55.37 (SD 8.22) at follow-up, indicating that participants scored half an SD above the mean of the US norm population (T-score: mean 50, SD 10). The mean FACT-G quality of life scores among participants was 75.48 (SD 13.89) at baseline and slightly reduced to 69.91 (SD 15.15) at follow-up. Although these scores are slightly lower than the mean among the norm population, both are within 1 SD of the mean.

**Table 2:** Mean scores for patient-reported outcomes at baseline and 12 weeks.

Outcome	Baseline (N=25), mean (SD)	12 weeks (n=23), mean (SD)
Self-efficacy for coping with cancer (CBI-B^a^)	84.44 (14.66)	75.74 (14.43)
Quality of life (FACT-G^b^)	75.48 (13.89)	69.91 (15.15)
Self-efficacy for managing emotions (PROMIS-SE^c^), T-score^d^	49.09 (8.21)	48.64 (7.80)
Perceived emotional support (PROMIS-ES^e^), T-score	55.24 (7.11)	55.37 (8.22)

^a^CBI-B: Cancer Behavior Inventory-Brief Version.

^b^FACT-G: Functional Assessment of Cancer Treatment-General.

^c^PROMIS-SE: Patient-Reported Outcomes Measurement Information System Self-Efficacy for Managing Emotions-Short Form.

^d^For T-scores: in the US norm population, the mean T-score is 50 (SD 10). Higher T-scores represent more of the concept being measured.

^e^PROMIS-ES: Patient-Reported Outcomes Measurement Information System Emotional Support-Short Form.

## Discussion

### Principal Findings

We developed and pilot-tested an intervention to facilitate an engaged community and learn coping skills among young adults with cancer. In this study, though the intervention was deemed acceptable and overall rated positively by participants, we failed to demonstrate feasibility of the *iaya* app intervention in its present form based on the number of log-ins and coping exercises used. Importantly, our feasibility criteria did not include engagement with the community feed, which was cited by participants as the most interesting and used feature of the app. Furthermore, although self-efficacy for coping with cancer and overall quality of life slightly decreased over time, scores for self-efficacy with regard to emotions and perceived emotional support remained largely stable. Overall, scores were relatively high for all patient-reported outcomes, reflecting the nonclinical nature of our sample.

The finding that user engagement was low, with only 9% (2/23) of participants meeting our combined feasibility criteria of log-ins and coping exercises used, is surprising in the context of ubiquitous smartphone use among the overall young adult population [12,13]. However, selective use and poor retention have been documented for mental health apps, with only a small portion of users using the apps for a long period [35,36]. Alternatively, our a priori feasibility criteria may not have been sufficiently comprehensive, as we did not include all features of app engagement in our definition. At this early stage, we were mostly interested in studying the overall feasibility of implementing a newly developed smartphone app intervention among a novel population. Defining meaningful engagement, or even feasibility, remains a challenge for mental health apps, which has also been described by other investigators, and there is a need to better define and measure engagement in these apps [37].

At least two characteristics of our study may explain why we found low use of our specific app intervention for log-ins and coping exercises used. First, we did not screen for unmet needs in the areas of coping with cancer or managing emotions, and our baseline data indicate that our sample scored relatively well on these domains, which may explain the lack of engagement with coping exercises in our nonclinical sample. Indeed, in the qualitative exit interviews, several patients stated that they did not use the coping exercises section because they felt they either did not need it or had other resources to help them cope with their cancer. In future studies, researchers should consider using the CBI-B as a screener to identify patients who need psychosocial services [28]. Second, our intervention was a stand-alone app with no external factors to motivate young adults with cancer to use it, except for phone reminders early in the first 3 weeks of enrollment and a midstudy call at 6 weeks. Although patients had the option to enable or disable app notifications, in exit interviews, the majority indicated turning off notifications per their personal preference. Although the app was not designed to be integrated within their larger clinical care, some patients indicated that they may have used it more frequently if a clinician encouraged them to use different features of the app or recommended specific elements. In line with the findings from a recent meta-analysis, it seems that despite the potential of apps, using smartphone apps as stand-alone psychological interventions cannot be recommended yet based on the current level of evidence [38]. Therefore, future studies should consider giving participants more directions on how to use the coping exercises and/or integrate the app within their larger clinical care.

Notwithstanding the low use, patients liked the app, deeming it acceptable and easy to use, and they only observed a few technical issues. The main suggestion for further improvement of the app was to increase the number of active users to facilitate greater community engagement and to update content on the app more frequently. We tried to mitigate low user engagement by soliciting *super users* to frequently post content and respond to other users’ contributions. In addition, we tried to build an active young adult community by recruiting patients to the app as part of clinical care 6 months before opening our research study for accrual. Between April and November 2019, we enrolled 104 patients outside of the research study. During the 5-month study enrollment period, another 107 patients downloaded the app outside of the research study, resulting in a total of 236 users. However, the number of contributing users overall was still relatively low for a microsocial network app, and many of the research participants who commented on the low user engagement on the app also admitted to adopting more of an observer role on the app, which may also generalize to the larger user base.

At the same time, making the app available before starting our research study presented us with a challenge, as our institutional review board required that we explicitly inform potential study participants that the app was freely available to them outside of the research study, disincentivizing study participation. This also contributed to our low enrollment rate, as 18% (12/65) of approached patients who declined study participation said they would rather download the app separate from the study. One potential solution would be to open up the app to a larger population, including other cancer centers in the Northeast region of the United States, or even nationwide. This would also increase the opportunities for patients to connect with a peer who has been diagnosed with a similar diagnosis, a desire that came up several times during the exit interviews and has been reported previously [16]. This need is understandable and also challenging given the rarity of many cancer diagnoses among young adults.

### Challenges

We encountered several challenges in this pilot feasibility study. First, one of our participants dropped out in the first 3 weeks of the study and did not engage in the study procedures. Second, one participant had an older version of the smartphone app installed, which was incompatible with Mixpanel. In addition, if study participants got a new phone during the study period, their earlier use data had to be merged with a new Mixpanel user ID; thus, we had to actively inquire whether participants got a new phone. Third, because of Health Insurance Portability and Accountability Act regulations, the app required participants to log in with their username and password each time they opened the app, even if they briefly switched to another app or function on their smartphone. These clustered log-ins were counted as 1 individual app session. Fourth, although we planned to conduct reminder phone calls to those patients who had not used the app for 14 consecutive days, we were unable to analyze and act upon these data in real time. Therefore, we changed this procedure to a midstudy reminder phone call at 6 weeks. Finally, 5 participants completed the study surveys more than 14 days after completion of their 12-week study period (ie, between 17 and 24 days).

### Limitations

Our study had some limitations. This was a pilot study conducted at a single site. Recruitment was suspended 2 months earlier because of the COVID-19 pandemic, and we had a slightly smaller sample size than anticipated. Although we enrolled a clinically diverse sample of 25 patients with different diagnoses, most were well educated, and our sample lacked racial and ethnic diversity. Importantly, as a feasibility pilot, the study did not have a control arm, and thus, we were unable to test the efficacy of the intervention.

### Conclusions

In conclusion, although considered acceptable, the *iaya* app did not meet the feasibility criteria we had originally posited, and user engagement was generally low. We gained valuable insights from our qualitative interviews and learned that participants highly valued the community feed aspect, which was not part of our a priori feasibility criteria. Future studies should consider targeting a clinical population with unmet needs, who may benefit more from the coping skills feature. The smartphone app intervention in its present form requires further adaption and refinement before conducting a larger, multisite, randomized clinical trial to assess the efficacy of the *iaya* app intervention on young adults’ self-efficacy for coping with cancer and managing emotions, perceived emotional support, and quality of life.

## Abbreviations

CBI-B: Cancer Behavior Inventory-Brief Version
FACT-G: Functional Assessment of Cancer Therapy-General
PROMIS: Patient-Reported Outcomes Measurement Information System
REDCap: Research Electronic Data Capture
